# The ICD-9 to ICD-10 transition has not improved identification of rapidly progressing stage 3 and stage 4 chronic kidney disease patients: a diagnostic test study

**DOI:** 10.1186/s12882-024-03478-1

**Published:** 2024-02-14

**Authors:** Kabir Jalal, Andre Charest, Xiaoyan Wu, Richard J. Quigg, Shirley Chang

**Affiliations:** 1 Department of Biostatistics, University at Buffalo, State University of New York, 807 Kimball Tower, 14214-3000 Buffalo, NY USA; 2 Division of Nephrology, Department of Medicine, University at Buffalo, Buffalo, USA

**Keywords:** Progression, CKD, ICD, Sensitivity, Specificity

## Abstract

**Background:**

The International Classification of Diseases (ICD) coding system is the industry standard tool for billing, disease classification, and epidemiology purposes. Prior research has demonstrated ICD codes to have poor accuracy, particularly in relation to rapidly progressing chronic kidney disease (CKD) patients. In 2016, the ICD system moved to revision 10. This study examines subjects in a large insurer database to determine the accuracy of ICD-10 CKD-staging codes to diagnose patients rapidly progressing towards end-stage kidney disease (ESKD).

**Patients and methods:**

Serial observations of outpatient serum creatinine measurements from 2016 to 2021 of 315,903 patients were transformed to estimated glomerular filtration rate (eGFR) to identify CKD stage-3 and advanced patients diagnosed clinically (eGFR-CKD). CKD-staging codes from the same time period of 59,386 patients and used to identify stage-3 and advanced patients diagnosed by ICD-code (ICD-CKD). eGFR-CKD and ICD-CKD diagnostic accuracy was compared between a total of 334,610 patients.

**Results:**

5,618 patients qualified for the progression analysis; 72 were identified as eGFR rapid progressors; 718 had multiple codes to qualify as ICD rapid progressors. Sensitivity was 5.56%, with positive predictive value (PPV) 5.6%. 34,858 patients were diagnosed as eGFR-CKD stage-3 patients; 17,549 were also diagnosed as ICD-CKD stage-3 patients, for a sensitivity of 50.34%, with PPV of 58.71%. 4,069 patients reached eGFR-CKD stage-4 with 2,750 ICD-CKD stage-4 patients, giving a sensitivity of 67.58%, PPV of 42.43%. 959 patients reached eGFR-CKD stage-5 with 566 ICD-CKD stage-5 patients, giving a sensitivity of 59.02%, PPV of 35.85%.

**Conclusion:**

This research shows that recent ICD revisions have not improved identification of rapid progressors in diagnostic accuracy, although marked increases in sensitivity for stage-3 (50.34% vs. 24.68%), and PPV in stage-3 (58.71% vs. 40.08%), stage-4 (42.43% vs. 18.52%), and stage-5 (35.85% vs. 4.51%) were observed. However, sensitivity in stage-5 compares poorly (59.02% vs. 91.05%).

## Background

The International Classification of Diseases (ICD) coding system is widely utilized for administrative, clinical, and epidemiological purposes. ICD codes serve a vital role in informing the medical community as key decisions are made regarding policy and reimbursement decisions [1]. On October 1, 2015, the 10th revision of the ICD coding system was implemented under mandate of the United States Department of Health and Human Services [2]. Previous research has examined the accuracy of ICD-10 coding with regard to Chronic Kidney Disease (CKD), but limited longitudinal data precluded examining ICD-10 coding data accuracy in the context of disease progression [3, 4]. This study utilizes ICD-10 data originating in a large claims database from 2016 to 2021 to assess ICD-10 coding accuracy among CKD patients.

The previous ICD-9 system was revised to ICD-10 with the aim of increasing specificity of the codes. This increased specificity allows for rapid incorporation of emerging diseases and higher detail allowing for more precise diagnostic codes. Consequentially, ICD-10 boasts 69,823 codes compared to only 14,025 for ICD-9 [2]. However, CKD diagnostic codes have not benefitted from the improvements from ICD-9 to ICD-10. Indeed, the primary diagnostic codes indicating CKD staging simply change the prefix from 585 to N18, yet continue to identify only the primary stages with no distinction between stage 3a and stage 3b. Codes indicating an underlying cause of CKD have increased allowing for more detailed diagnosis and better tracking of the disease’s etiology, though whether this translates to improved diagnostic has not been established.

Studies of agreement between ICD-9 coding and gold-standard clinical markers have demonstrated disease-dependent accuracy rates. Cardiovascular diseases, stroke, and pneumococcal pneumonia, for example, have all been shown to have accurate ICD codes [5–7]. Similar studies with ICD-10 data have drawn conclusions consistent with previous ICD-9 based research [8–10]. That these conditions generally present with clear symptoms may partially explain the accuracy of their related codes.

Chronic Kidney Disease (CKD) coding accuracy, however, is notably deficient, with many ICD-9 studies reporting low sensitivity rates with high specificity rates [3, 11, 12]. Meta-analyses and systematic reviews of the surrounding literature report widely varying sensitivity and specificity rates, suggesting inconsistent coding practices and accuracy [13, 14]. Research utilizing ICD-10 codes has not shown substantial improvement [3]. However, a recent study demonstrated that utilizing multiple CKD codes in conjunction may yield acceptable diagnostic accuracy [4]. These latest results notwithstanding, the subtle nature of CKD and its common presentation alongside other comorbid conditions may offer some cause for the poor diagnostic utility of ICD codes in identifying clinical CKD.

The identification of rapid progressors, defined as those with yearly estimated glomerular filtration rate (eGFR) loss greater than 4 ml/min/1.73 m²) would allow for expedient care for those suffering from advanced CKD. Our previous work showed that ICD-9 CKD staging codes and their use was insufficient to identify patients with rapidly progressing CKD [3]. However, only two years of ICD-10 data was available at the time of that prior study, and therefore progression analysis was not possible.

This manuscript expands our prior research and leverages five years of outpatient ICD-10 codes to evaluate coding accuracy along three objectives:

- Rapid Progression Accuracy: Rapidly progressing patients identified clinically using longitudinal eGFR were compared against patients with multiple ICD-10 CKD staging codes indicating increasing disease severity to determine accuracy of ICD-10 codes.
- Overall and Stage-Stratified Accuracy: CKD patients identified clinically using multiple eGFR measures were compared against those with any ICD-10 code indicating CKD to determine overall accuracy. Further, CKD patients were assigned a CKD stage based on eGFR measures and compared against those with ICD-10 CKD staging codes to assess accuracy of ICD-10 staging codes.
- Demographic/Comorbidity Varying Accuracy: Agreement of the two diagnostic paradigms (eGFR-based and coding-based) was modeled against demographic and comorbidity data in a multivariate logistic regression to assess if diagnostic accuracy improves with varying patient demographic and comorbid profiles.

## Methods

This study utilized claims data from a large third party insurer, servicing over 1.3 million patients across the Western New York and Albany areas of New York State. Consisting of ten years of data from 2011 to 2021, prior research has explored this rich database [3, 15]. Focusing on the five-year period from 2016 to 2021, this study examines ICD-10 coding accuracy in the context of CKD. Patients with stage-3 CKD were identified using measured serum creatinine values and estimated glomerular filtration rate (eGFR) using a modified eGFR formula to exclude race [16]. With unique patient identifiers and observation dates, these eGFR values were linked to diagnostic ICD codes.

Based on clinician interpretation of Kidney Disease Outcomes Quality Initiatives (KDOQI) guidelines, patients with serum creatinine, age, and gender had eGFR values calculated. Those with two eGFR measures less than 60 ml/min/1.73 m² at least ninety days apart, with no intervening measurement greater than 60 ml/min/1.73 m², were identified by their eGFR as stage-3, stage-4, or stage-5 CKD cases. Limited presence of lab values precluded albuminuria-based stage 1 and stage 2 CKD diagnosis. Individuals with laboratory-confirmed CKD are referred to as eGFR-CKD.

CKD patients were alternatively identified using ICD-10-CM codes. The following code groups were considered: Chronic Kidney Disease (N18.1, N18.2, N18.3, N18.4, N18.5, N18.6, N18.9), Hypertensive CKD and hypertensive heart and CKD (I12.0, I12.9, I13.0, I13.1, I13.10, I13.11, I13.2), and diabetic mellitus with CKD (E08.21, E08.22, E08.29, E09.21, E09.22, E09.29, E10.21, E10.22, E10.29, E11.21, E11.22, E11.29, E13.21, E13.22, E13.29). Patients with at least one occurrence of any code were classified as ICD-CKD.

A longitudinal mixed model analysis was used to estimate the rate of eGFR progression over time using the eGFR-CKD patients [17]. Patients were followed from initial entry into CKD-stage 3 until they reached CKD-stage 5, or end-stage-kidney-disease (ESKD) treatment was initiated. Only patients with at least three years of follow-up data and five observations were included. eGFR was modeled against fixed and random effects of time (measured in quarter-year increments), and a random intercept was also included in the model. Those patients who experienced a yearly loss of eGFR greater than 4 ml/min/1.73 m² were considered to be rapid progressors [18, 19].

Based on the mixed model, Estimated Best Linear Unbiased Predictors (EBLUPs) for each patient was calculated [20]. Based on the slope derived from the EBLUPs, each patient was categorized as rapid progressors (RP). For the ICD-CKD patients that also met inclusion criteria for the progression analysis, ICD-10 staging codes (N18.3, N18.4, N18.5) were used to identify RP. Those with at least two codes of increasing stage were considered as such. Thus, each patient in the analysis was categorized as an eGFR-RP or ICD-RP or not.

To assess the accuracy of ICD-CKD and ICD-RP to indicate eGFR-CKD and eGFR-RP, epidemiological quantities for sensitivity (#true positives/[#true positives + #false negatives]), specificity (#true negatives/[#true negatives + #false positives]), positive predictive value (PPV; #true positives/[#true positives + #false positives]) and negative predictive value (NPV; #true negatives/[#true negatives + #false negatives]) were estimated with 95% confidence intervals. These four quantities are referred to as “performance measures” in this paper.

Agreement of ICD- and eGFR-CKD diagnoses was modeled against gender, age > 65, and comorbid conditions (proteinuria, diabetes, congestive heart failure, other heart diseases, and hypertension) in a multivariate logistic regression. Receiver operating characteristic (ROC) curves were generated using the Mann-Whitney association to estimate the area under the curve (AUC). A non-informative curve with AUC of 0.5 was held as reference, and every other curve was compared using a non-parametric approach [21].

## Results

Of the approximately 1.3 million patients in the claims database, 336,752 had sufficient serum creatinine measurements to determine eGFR-CKD status. Of these, 21,328 patients were identified as eGFR-CKD and 48,322 were ICD-CKD. Table 1 summarizes the sample demographics and selected comorbidities. Results of McNemar’s test showed differences in proportions across all groups (*p* < 0.0001).

**Table 1:** Demographic summary

	Overall Sample (*N* = 336,752)	eGFR-CKD (*N* = 21,328)	ICD-CKD (*N* = 48,322)
	% Yes	% Yes	% Yes
Male Gender	44.94	41.05	50.23
Age > 65	19.98	57.05	34.9
Proteinuria	0.36	2.7	2.13
DM2	19.36	46.84	53.48
Hypertension	49.24	92.57	92.24
Congestive Heart Failure	6.01	28.12	26.58
Other Heart Issues	6.33	30.21	28.08
CVA/CVD	10.28	34.47	31.37
CAD	13.93	44.16	43.28
COPD	10.01	30.04	28.28
Asthma	14.16	14.76	14.67
	Progression Sample (*N* = 5,618)	eGFR-RP (*N* = 72)	ICD-RP (*N* = 718)
	% Yes	% Yes	% Yes
Male Gender	43.22	100	54.46
Age > 65	67.5	43.06	63.65
Proteinuria	1.5	0	6.27
DM2	27.64	37.5	34.26
Hypertension	54.31	72.22	49.44
Congestive Heart Failure	18	18.06	31.62
Other Heart Issues	19.49	22.22	33.98
CVA/CVD	27.93	31.94	36.91
CAD	23.37	22.22	28.27
COPD	19.92	20.83	24.65
Asthma	10.32	11.11	9.89

Of the 5,618 patients qualifying for the progression analysis, 72 were identified as eGFR-RP, while 718 had multiple codes to qualify as ICD-RP patients. However, only 4 of these patients were among the eGFR-RP. Sensitivity was 5.56% (1.53, 13.62), with PPV 5.6% (1.5, 14.2), and specificity 87.13% (86.22, 88.00), with NPV 98.61% (98.24, 98.92). Table 2 summarizes the progression analysis sample.

**Table 2:** Contingency table of eGFR-based identification against ICD identification of rapid progressors (RP)

		ICD-RP		
		Yes	No	Total
eGFR-RP	Yes	4 5.56%	68 94.44%	72 1.28%
	No	714 12.87%	4832 87.13%	5546 98.72%
	Total	718 12.78%	4900 87.22%	5618 100%

When considering all CKD codes as well as diabetic, hypertensive, and heart disease codes that also indicate CKD against eGFR-CKD status, ICD codes perform well, with a sensitivity of 77.12% (76.56, 77.68). Sensitivity for staging codes is varied, with a low of 50.41% (49.89, 50.94) among clinically identified stage-3 patients, to a high of 67.82% (66.39, 69.25) among stage-4 patients, and finally 60.62% (57.68, 63.56) among stage-5 patients. Full results can be seen in Table 3 below.

**Table 3:** Performance measures

	Overall Performance			
	Measure	Mean	Lower 95%	Upper 95%
	Sensitivity	77.12	76.56	77.68
	Specificity	89.89	89.79	90
	Positive Predictive Value	34.04	33.62	34.46
	Negative Predictive Value	98.31	98.26	98.36
	Stage-Stratified Performance			
	Measure	Mean	Lower 95%	Upper 95%
Stage 3	Sensitivity	50.41	49.89	50.94
	Specificity	95.9	95.83	95.97
	Positive Predictive Value	58.72	58.16	59.27
	Negative Predictive Value	94.35	94.27	94.43
Stage 4	Sensitivity	67.82	66.39	69.25
	Specificity	98.86	98.83	98.9
	Positive Predictive Value	42.4	41.2	43.59
	Negative Predictive Value	99.6	99.58	99.62
Stage 5	Sensitivity	60.62	57.68	63.56
	Specificity	99.67	99.65	99.69
	Positive Predictive Value	36.92	34.66	39.23
	Negative Predictive Value	99.87	99.86	99.89

**Fig. 1:**
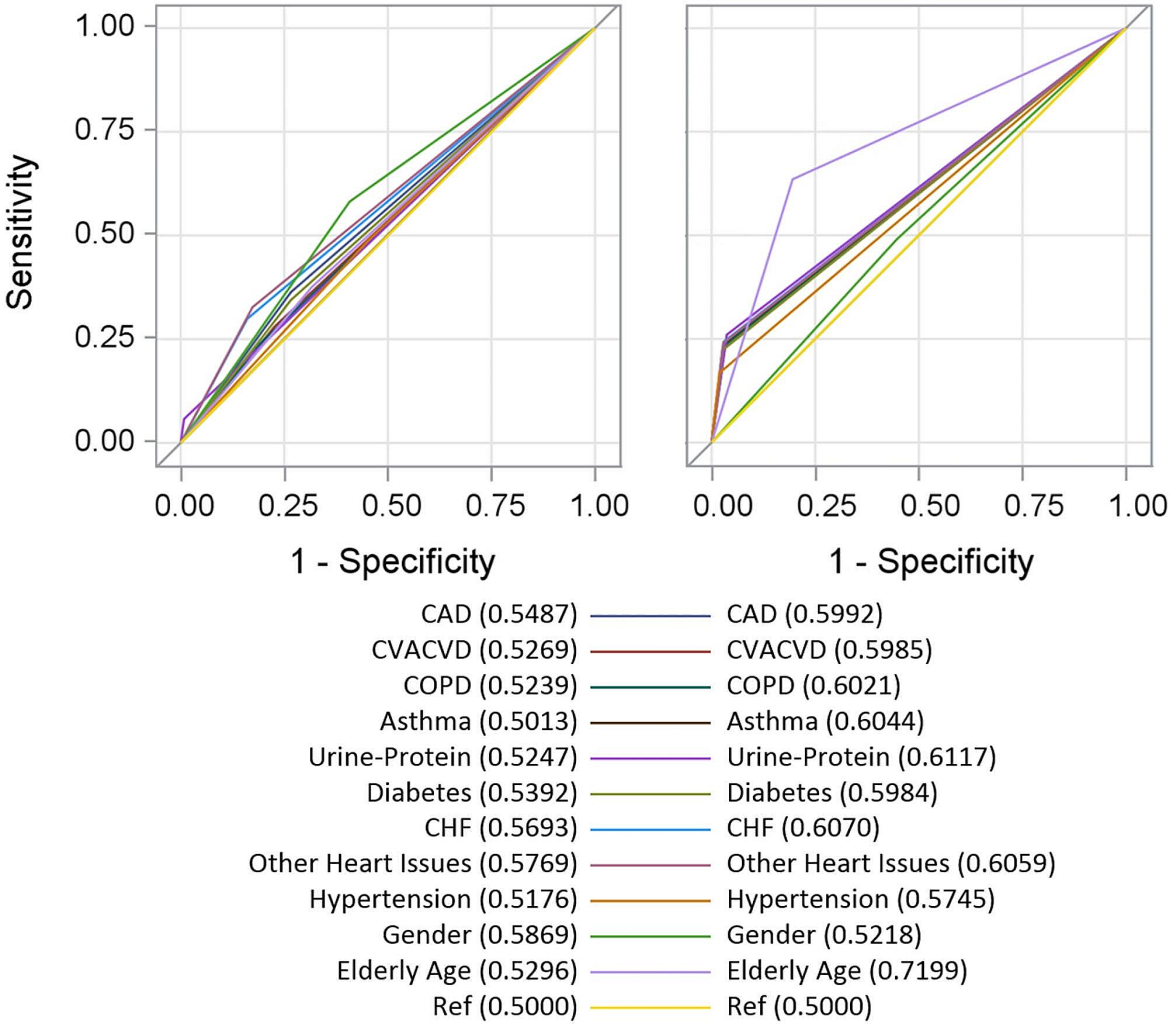
ROC curves for comorbidities in progression (left) and overall (right) samples

In the progression sample, ROC analysis showed little improvement in detection of rapid progressors when controlling for comorbid history, with heart issues offering the greatest advantage in predictive value over an arbitrary decision (AUC = 0.5769, 95% CI = 0.5596,0.5942). In the overall sample, minor to moderate improvement to overall coding accuracy compared to over an arbitrary decision when controlling for comorbidities. Elderly age (AUC = 0.7199, 95% CI: 0.7163, 0.7235) added the most predictive value. AUCs are plotted in Fig. 1 below.

## Discussion

Detection of individuals who are experiencing rapidly progressing CKD is a critical step in treatment. Utilization of ICD codes to programmatically identify potential rapid progressors would allow for expeditious care for those at the highest risk. This study is the first to explore the viability of ICD-10 codes and practices in detecting rapid progressors and CKD patients in general. As shown previously, ICD codes remain ineffective at either of these tasks [3].

While the CKD-staging codes identify the major stages of the disease, the ICD-10 revision has done little to mark the more subtle changes that may indicate a patient at risk for rapid progression. Compared to our previous work with ICD-9 data, diagnostic accuracy for RP patients was worse among most measures [3]. Sensitivity was 5.56% in the current ICD-10 study vs. 25.7% in the previous ICD-9 study, PPV 5.6% vs. 14.2%, specificity was 87.13% vs. 94.94%, with only NPV showing slight improvement at 98.61% vs. 97.73%.

An additional code to separate CKD-stage 3 into the commonly used stage 3a and stage 3b subtypes would perhaps improve detection rates for patients at this critical junction in their CKD course. This problem has been addressed in the upcoming ICD-11 revision, however, with distinct codes for stage 3a and stage 3b included [22].

Table 4 below summarizes selected research studies into coding accuracy.

**Table 4:** Characteristics of studies on diagnostic accuracy of chronic kidney disease

Reference	Location	Population Selection Criteria	Study Timeframe	Sample Size	Gold-Standard Definition of Kidney Disease	Diagnostic Tool for Kidney Disease	Sensitivity & Specificity	Additional Notes
*Current Study*	Western New York	Outpatient data with two valid serum creatinine	2016–2021	315,903	KDOQI based on eGFR w/o race	29 ICD-10 Codes	50.3, 95.88	Gold-Standard based on 2 eGFR measures
Paik, 2021 [4]	Harvard Medical School	Outpatient lab values	2016–2018	373,220	Lab-based eGFR within pre-specified windows	3 ICD-10 Codes	-	PPV > 80%
Ko, 2018 [23]	Melbourne, Australia	One eGFR < 60	2012	325	KDIGO based on one eGFR	44 ICD-10 Codes	54.1, 90.2	-
Jalal, 2019 [3]	Western New York	Outpatient data with two valid serum creatinine	2007–2014, 2016–2017	216,529	KDOQI based on CKD-EPI eGFR	27 ICD-9 Codes, 7 ICD-10 Codes	32.2, 97.12	Gold-Standard based on 2 eGFR measures
Chase et al. 2010 [24]	Columbia University Medical Center	Outpatient data with two elevated serum creatinine values	2003–2006	175	KDOQI based on CKD-MDRD eGFR	Electronic Health Records containing CKD documented in notes	95.4–99.8 & 99.8	All hypertensive patients
Ronksley 2012 [25]	Alberta, Canada	Outpatient with two elevated serum creatinine values	2004–2005	321,293	KDOQI based on CKD-MDRD eGFR	25 ICD-9 Codes	18.9–29.3 & 94.6–98.5	Gold-Standard based on 2 eGFR measures
Cipparone 2015 [11]	Buffalo, Kansas	Inpatient Chart Review	-	325	Chart review protocol based on KDOQI Guidelines	ICD-9 585.3 Code	-	Prevalence of misdiagnosis; no Sensitivity or Specificity
Fleet 2013 [12]	Ontario, Canada	Outpatient age > 65	2007–2010	123,499	CKD-EPI eGFR < 60; < 45; < 30	Algorithm of hospital encounter and 11 ICD-9 Codes	18 & 98.2	Gold-Standard based on only 1 eGFR measure
Winkelmayer 2005 [26]	Pennsylvania	Medicare Inpatients	1999–2000	1,852	CKD-MDRD eGFR < 60	22 ICD-9 Codes	2–27 & 93–100	Gold-Standard based on only 1 eGFR measure
Kern 2006 [27]	US VA and Medicare Systems	Inpatient and Outpatient Diabetics in VA System	1999–2000	263,730	CKD-MDRD eGFR < 60	79 ICD-9 Codes	20–41 & 95–99	Gold-Standard based on only 1 eGFR measure
Stevens 2005 [28]	Laboratory Corporation of America, Columbus, OH	Outpatient age > 39	2002–2003	277,111	CKD-MDRD eGFR < 60	51 ICD-9 Codes	10–51 & 95–98	Gold-Standard based on only 1 eGFR measure
Navaneethan 2011 [29]	Cleveland Clinic Patients	Outpatient with two elevated serum creatinine values and/or two ICD-9 diagnoses	2005–2010	296,249	KDOQI based on CKD-MDRD eGFR	8 ICD-9 Codes	> 80	Gold-Standard based on 2 eGFR measures
Lardon 2015 [30]	French PMSI Hospitals	Inpatient age 12–65 or 80	January, 2014	533	eGFR	Drools rules engine based on EHR and ICD-10	-	Analyzed hospital stays, rather than patients

Compared to our previous study on ICD-9 data, the ICD-10 codes utilized in this study have shown improvement in sensitivity for stage-3 (50.34% vs. 24.68%), and PPV in stage-3 (58.71% vs. 40.08%), stage-4 (42.43% vs. 18.52%), and stage-5 (35.85% vs. 4.51%). However, sensitivity in stage-5 compares poorly (59.02% vs. 91.05%) [3]. Other ICD-10 studies have shown similar performance [23]. Novel approaches that combine multiple codes may yield improvement [4].

Comparing diagnostic accuracy using any qualifying code showed improved sensitivity (77.12% vs. 32.16%) and NPV (98.31% vs. 90.33%), but worse PPV (34.04% vs. 63.10%) and specificity (89.89% vs. 97.12%) [3]. These mixed results of the diagnostic accuracy measures may reflect the increased amount of secondary codes indicating underlying CKD causes.

Generally speaking, ICD-10 coding appears to have some accuracy improvement over ICD-9. Given the similarity between ICD-9 and ICD-10 coding, it is likely that this improvement is derived from clinical practices. Increased reliance on electronic health records (EHR) and physicians becoming more facile with current technologies, as hospital administrators and staff implement policies to comply with EHR mandates. EHR implementation has been criticized for disrupting workflow and increasing workload, although positive effects of increased data collection has been seen over time [31]. Improved diagnostic accuracy of ICD codes may be a result of this changing paradigm.

This study has limitations, largely related to the nature of claims data. Chief among them is the lack of racial data. While this demographic variable is not present in the formulation of eGFR used here, racial disparities are commonplace in medicine, and these results may be subject to this phenomenon [16]. Additionally, these data are derived from privately insured patients in the United states and may not be reflective of patient experiences or caregiver practices with respect to ICD coding in other countries.

## Conclusion

The study presented here has utilized claims data from patients followed from 2016 to 2021, and it demonstrates that coding accuracy has not improved substantially since adoption of the ICD-10 coding standards in the context of CKD. There remains a gulf between clinically derived diagnostic procedures and attempts at ICD-based diagnosis. Consequentially, clinical markers remain the only viable tool for identifying CKD patients, rapidly progressing or otherwise. Future work may include attempts to utilize multiple codes in concert to increase diagnostic accuracy.

## Data Availability

The datasets generated during and analyzed during the current study are not publicly available due to their licensed use for the current research but are available from the corresponding author, Kabir Jalal, on reasonable request.

## Abbreviations

AUC: Area Under the Curve
CKD: Chronic Kidney Disease
ESRD: End-Stage Renal Disease
eGFR: estimated Glomerular Filtration Rate
ICD: International Classification of Diseases
KDOQI: Kidney Disease Outcomes Quality Initiative
NPV: Negative Predictive Value
PPV: Positive Predictive Value
ROC: Receiver Operator Characteristic
